# Efficacy and Safety of Oxymetazoline 1% Cream for the Treatment of Mild to Moderate Facial Rosacea

**DOI:** 10.1111/jocd.16782

**Published:** 2025-04-01

**Authors:** Fatemeh Sajdeh, Aniseh Samadi, Atefeh Naeimifar, Taraneh Yazdanparast, Maryam Ahmadi, Fatemeh Amiri, Martin Kassir, Alireza Firooz, Saman Ahmad Nasrollahi

**Affiliations:** ^1^ Center for Research and Training in Skin Diseases and Leprosy Tehran University of Medical Sciences Tehran Iran; ^2^ Worldwide Laser Institute Dallas Texas USA; ^3^ Clinical Trial Center Tehran University of Medical Sciences Tehran Iran

**Keywords:** clinical trial, oxymetazoline, rosacea, skin biometric parameters

## Abstract

**Introduction:**

Rosacea is a common skin condition that affects the physical and mental health of the patient. The objective of this study was to evaluate the efficacy and safety of oxymetazoline 1% cream, a specific α1A‐adrenoceptor agonist, for the treatment of mild to moderate facial rosacea.

**Methods:**

In phase II of the before–after study, 15 patients with mild to moderate rosacea were enrolled and treated with oxymetazoline 1% cream twice daily for 4 weeks. The clinician's erythema assessment (CEA) and patient's self‐assessment (PSA), as well as the skin biometric parameters (transepidermal water loss, sebum, stratum corneum hydration, temperature, erythema, melanin, and pH), dermal and epidermal thickness and echo‐density, and capillaroscopic pattern were assessed and compared before and after treatment. *p*‐Value < 0.05 was considered significant.

**Results:**

CEA and PSA indexes decreased significantly 2 and 4 weeks after the treatment (*p*‐value: 0.001). The erythema index also decreased significantly in both 2 weeks (*p*‐value: 0.001) and 4 weeks (*p*‐value: 0.001) after the treatment. The capillaroscopic pattern of lesions showed a decrease in telangiectasia and a reddish background of skin. No side effects of treatment were experienced by any of the patients.

**Conclusion:**

Oxymetazoline 1% cream was safe and effective in treating mild to moderate facial rosacea. The reduction in the skin's vascularity was also objectively confirmed.

## 1 Introduction

Rosacea is a chronic inflammatory disease of the central facial skin with an unknown cause, which affects the physical and mental health of the patient [1]. It is characterized by transient erythema or flushing, which may become persistent and lead to papules and pustules, hyperplasia of the connective tissue, and telangiectasia [2, 3]. Less common findings related to the symptoms include erythematous plaques, dryness and scaling, phymatous changes, edema, extra‐facial location, and ocular symptoms [4]. The pathophysiology of rosacea is not fully understood. It seems that dysregulation of adaptive and innate immune systems and neurovascular changes predispose individuals to develop abnormal, sustained vasodilation in the facial skin [5].

The most frequent and consistent feature of rosacea is facial erythema, which is also the most difficult one to treat [6]. Many people experiencing facial erythema due to rosacea are embarrassed by the condition and feel that they are stigmatized by society. The problem of the negative impact of rosacea on quality of life is still underestimated, and effective treatment of rosacea‐associated facial erythema is exceedingly important to patients [7].

Despite advancing knowledge on rosacea pathogenesis, diagnosis, and treatment, systematic and evidence‐based guidelines useful for therapeutic management are still lacking, although this knowledge has led to the development of new therapeutic agents [8]. Topical treatment is the first‐line option for mild to moderate forms of the disease. The main need in topical treatment is the management of vasomotor flushes and telangiectasia [8].

Topical alpha‐adrenergic receptor agonists are effective in decreasing facial erythema [9]. Oxymetazoline hydrochloride is an α_1_A adrenoceptor agonist and has a vasoconstrictor role in skin microvasculature [10]. It is recommended for the topical treatment of persistent facial erythema associated with rosacea in adults and was approved by the US FDA in 2017 [11]. But, to our knowledge, no published study evaluates the effect of oxymetazoline on the biophysical characteristics of the skin in rosacea patients, while it is proven that skin biometric assessment is one of the most accurate means for evaluating the skin [12, 13, 14].

The purpose of this study was to evaluate the clinical efficacy and safety of oxymetazoline cream 1% for the treatment of mild to moderate facial rosacea in a group of Middle Eastern patients, as well as its effects on skin biophysical parameters.

## 2 Method

This was a phase II, before–after clinical trial on patients with mild to moderate facial rosacea who were referred to the Center for Research and Training in Skin Diseases and Leprosy (CRTSDL) at the Tehran University of Medical Sciences in Tehran, Iran from May to October 2020.

This study was approved by the ethics committee of the Tehran University of Medical Sciences (code IR.TUMS.VCR.REC.1397.720). The research was conducted following the Declaration of Helsinki and the principles of Good Clinical Practice. Patients were recruited into the study based on the inclusion and exclusion criteria, and they were informed of the study protocol and signed the written informed consent. The participants also provided consent for the publication of any pictures.

Inclusion criteria consisted of being 18–50 years of age and having mild to moderate rosacea, as diagnosed by a dermatologist based on clinical findings and according to a Clinician's erythema assessment (CEA) score of 3–4, and having otherwise a normal systemic health status. Exclusion criteria included the use of systemic corticosteroids, tetracycline, and isotretinoin within 4 weeks before the study; use of antihistamines within 3 months before the study; use of topical exfoliating products such as AHA, BHA, and retinoid compounds within 3 weeks before the study; use of topical corticosteroids, oxymetazoline, ivermectin, brimonidine, and tetracycline before the initiation of the study; active skin diseases such as atrophy, acne, eczema, or scars at the test area; allergy to oxymetazoline or any excipient of the cream; laser treatment, dermabrasion, or deep facial peeling within 3 months before the study; and pregnancy or lactation.

Baseline assessments were performed at the first visit (before intervention). The participants were instructed not to use any topical products from the night before the assessments. Before measurements, the participants were asked to rest in a supine position for 20 min in a standard atmosphere (21° ± 2°C and 40% ± 5% humidity). Skin biophysical parameters including transepidermal water loss (TEWL), sebum, stratum corneum hydration, temperature, erythema & melanin index, and pH were measured by the Tewameter TM 300, Sebumeter SM 815, Corneometer CM 825, Skin‐Thermometer ST 500, Mexameter MX 18, and Skin‐pH‐Meter PH 905 probes of CutometerMPA 580 (CK Company, Cologne, Germany).

Also, the thickness and echo density of the epidermal and dermal layers were measured using a 22 MHz probe of a high‐frequency ultrasound digital apparatus (DUB‐USB skin scanner, tpm Co. Luneburg, Germany).

Visioface digital photography imaging system (CK Company, Cologne, Germany) was utilized for the evaluation of facial skin, consisting of a cabin attached to a high‐resolution digital camera (10 megapixels) and 200 white LEDs. The images were analyzed using the VisioFace CSI software. The Δ*L* score—a parameter that classifies the sensed color difference—and the Δ*E* score—a parameter that classifies the sensed erythema difference—were calculated. The final Δ*E* and Δ*L* scores were the difference of 1 point on the most severe lesion from a comparison point on the nearest healthy skin in the sagittal plane.

The capillaroscopic pattern of lesions was assessed by FotoFinder Medicam 1000 video dermatoscope with the use of a 20‐fold magnification. For wet video dermoscopy, a local ultrasound gel was used, and the vasculature of the lesions was assessed with minimal pressure to avoid blanching.

All these aforementioned measurements were performed on the forehead, cheek, or chin, depending on the severity of the lesions in each area. The location of the most severe lesions was selected for assessment and marked on case report forms.

Clinical erythema severity was assessed by the Clinician's erythema assessment (CEA) and Patient's Self‐Assessment (PSA) scales. Both scores were recorded using 5‐point scales and are shown in Table 1. The CEA score was evaluated by two blinded dermatologists, and an average score was analyzed.

**TABLE 1:** Clinician's erythema assessment (CEA) and patient's self‐assessment (PSA) scales.

Score	Clinician's erythema assessment (CEA)	Patient's self‐assessment (PSA)
0	Clear skin with no signs of erythema	No redness
1	Almost clear, slight redness	Very mild redness
2	Mild erythema, definite redness	Mild redness
3	Moderate erythema plus marked redness	Moderate redness
4	Severe erythema plus fiery redness	Severe redness

The test product was oxymetazoline hydrochloride 1% as an O/W cream, produced by Janus Pharmaceutical Co, Tehran, Iran. Participants were instructed to wash their skin with mild cleansers and then apply a thin layer of the cream to areas with erythema, every night. After 1 week, if there were no symptoms of skin allergies, the cream was used every night, 30 min before bedtime, for another 3 weeks. The participants were also instructed to regularly apply a mild moisturizer and broad‐spectrum sunscreen with SPF 30 or more, every day.

All measurements were repeated on the same area as baseline 2 and 4 weeks after treatment. In order to evaluate the safety and tolerability of the tested cream, the subjects were asked to record any dryness, itching, stinging, redness, scaling, or pigmentation changes during the treatment. In case of any severe adverse effects, the area was photographed, and if the participant could not continue the study, the use of the cream was stopped, and the measurements were done on the same or the next day.

Statistical analysis was performed using SPSS Software version 20.0 (IBM, USA). Skin parameters at weeks 2 and 4 were compared with baseline values using a paired‐samples *t*‐test. *p*‐Values less than 0.05 were considered statistically significant.

## 3 Results

Fifteen patients (12 females and 3 males) with mild to moderate rosacea were enrolled in the study. The age range was 18–50 years. All patients completed the trial.

**FIGURE 1:**
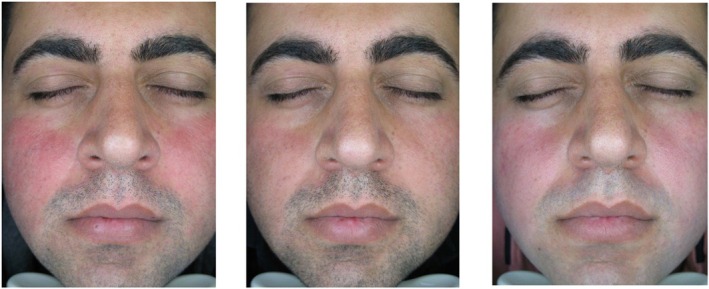
Erythema decrease in a rosacea patient after 2 and 4 weeks of treatment with oxymetazoline cream 1%.

Table 2 shows the comparison of skin biophysical and ultrasonographic parameters before, 2 weeks, and 4 weeks after the treatment. According to the table, the erythema index decreased significantly at both two (*p*‐value: 0.001) and 4 weeks (p‐value: 0.001) after the treatment. Erythema decrease was also seen in the patients' photos taken by the Visioface digital photography imaging system (Figure 1). Stratum corneum hydration decreased significantly in visit 2 (*p*‐value: 0.02), but this decrease wasn't significant in visit 3 (*p*‐value: 0.65). Other skin biophysical and ultrasonographic properties showed no significant difference before and after the treatment.

**TABLE 2:** Comparison of skin biophysical and ultrasonographic parameters before, 2 weeks, and 4 weeks after treatment with oxymetazoline cream 1%.

Skin biophysical parameters (Unit)	Visit 1 (before treatment) Mean ± SD	Visit 2 (after 2 weeks) Mean ± SD	Visit 3 (after 4 weeks) Mean ± SD	*p*‐Value 1 (Visit 1 and 2)	*p*‐Value 2 (Visit 1 and 3)
TEWL (g/m^2^/h)	26.96 ± 13.02	29.73 ± 18.48	23.77 ± 10.84	0.55	0.37
Sebum (μg/cm^2^)	39.33 ± 35.22	28.06 ± 25.66	36.80 ± 27.42	0.10	0.79
Hydration (arbitrary)	62.68 ± 13.18	50.54 ± 19.64	60.73 ± 13.43	**0.02**	0.65
Temperature (centigrade)	30.83 ± 1.36	30.50 ± 1.58	30.91 ± 1.70	0.50	0.88
Erythema index (arbitrary)	610.55 ± 125.53	501.42 ± 110.22	508.97 ± 101.49	**0.001**	**0.001**
Melanin index (arbitrary)	188.39 ± 65.42	170.22 ± 63.23	188.77 ± 58.87	0.25	0.97
pH (arbitrary)	5.90 ± 0.47	6.14 ± 0.41	6.01 ± 0.51	0.09	0.47
Epidermal thickness (μm)	106.26 ± 21.42	96.93 ± 22.17	97.06 ± 14.20	0.21	0.15
Epidermal density (arbitrary)	138.98 ± 30.25	145.27 ± 12.44	152.04 ± 13.77	0.51	0.23
Dermal thickness (μm)	1508.33 ± 496.18	1598.86 ± 426.44	1457.20 ± 364.05	0.15	0.68
Dermal density (arbitrary)	24.35 ± 7.23	23.42 ± 7.63	24.93 ± 7.61	0.64	0.75

*Note:* Bold values are statistically significant.

Abbreviation: SD, standard deviation.

**FIGURE 2:**
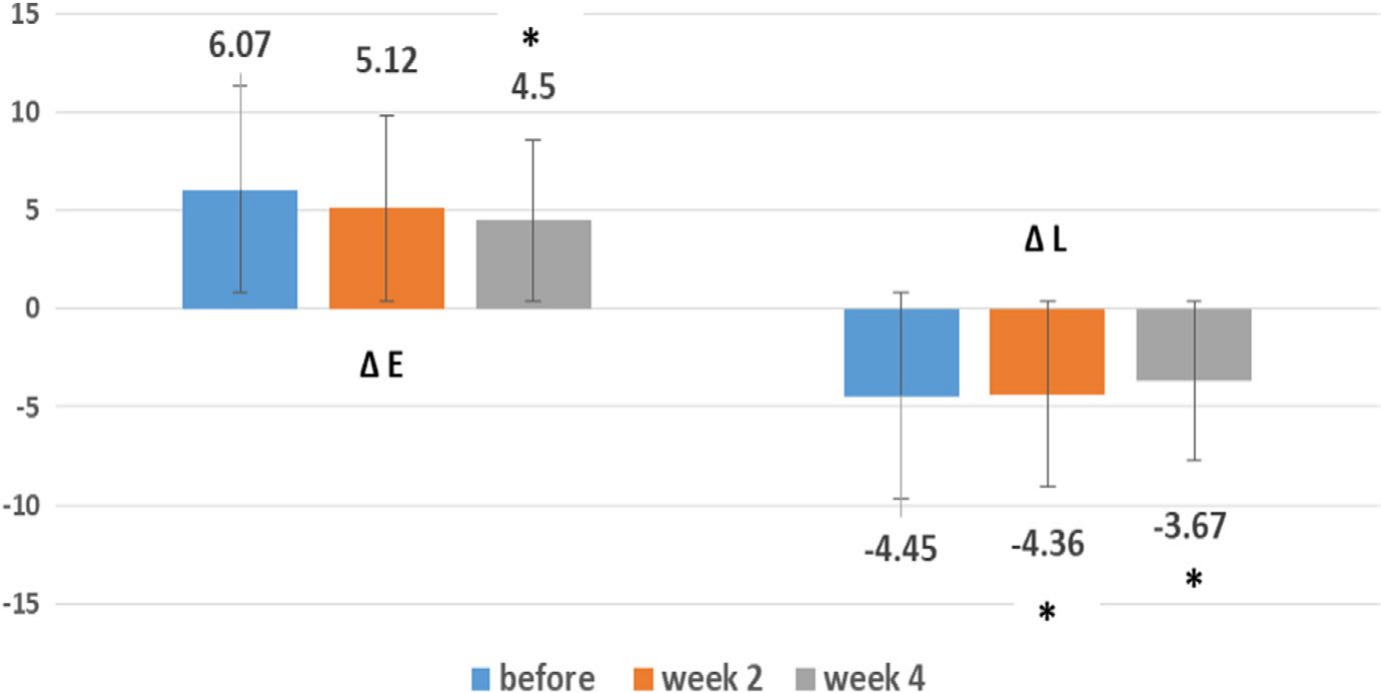
Δ*E* decrease after 2 and 4 weeks, and Δ*L* increase after 2 and 4 weeks of treatment with oxymetazoline cream 1% in rosacea patients (*p*‐value < 0.05). Δ*E*: erythema difference between lesion and healthy skin. Δ*L*: lightness difference between lesion and healthy skin.

Baseline Δ*E* or erythema difference between the lesion and healthy skin (mean: 6.07, SD: 2.25) was decreased after 2 weeks (mean: 5.12, SD: 1.76) and also after 4 weeks (mean: 4.50, SD: 1.99) of treatment. Comparing the first and second visits, the difference was not significant (*p*‐value: 0.20), and comparing the first and third visits, the difference was significant (*p*‐value: 0.04) (Figure 2).

Baseline Δ*L* or lightness difference between the lesion and healthy skin (mean: −4.45, SD: −1.83) was increased after 2 weeks (mean: −4.36, SD: −0.50) and also after 4 weeks (mean: −3.67, SD: −0.34) of treatment. This difference between the first and second visit, and also the first and third visit, was significant (*p*‐value: 0.02 and 0.03 respectively) (Figure 2).

**FIGURE 3:**
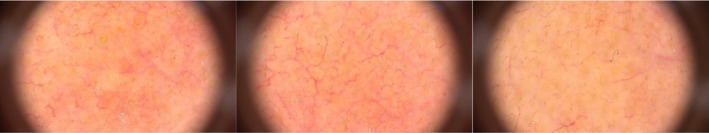
Telangiectasias and vessel diameter decrease in rosacea patients after 2 and 4 weeks of treatment with oxymetazoline cream 1%.

The capillaroscopic pattern of lesions, assessed by FotoFinder Medicam 1000 video dermatoscope, showed that telangiectasias and vessel diameter decreased after the treatment. Moreover, the reddish background, due to subpapillary vessel dilation, became paler (Figure 3).

**FIGURE 4:**
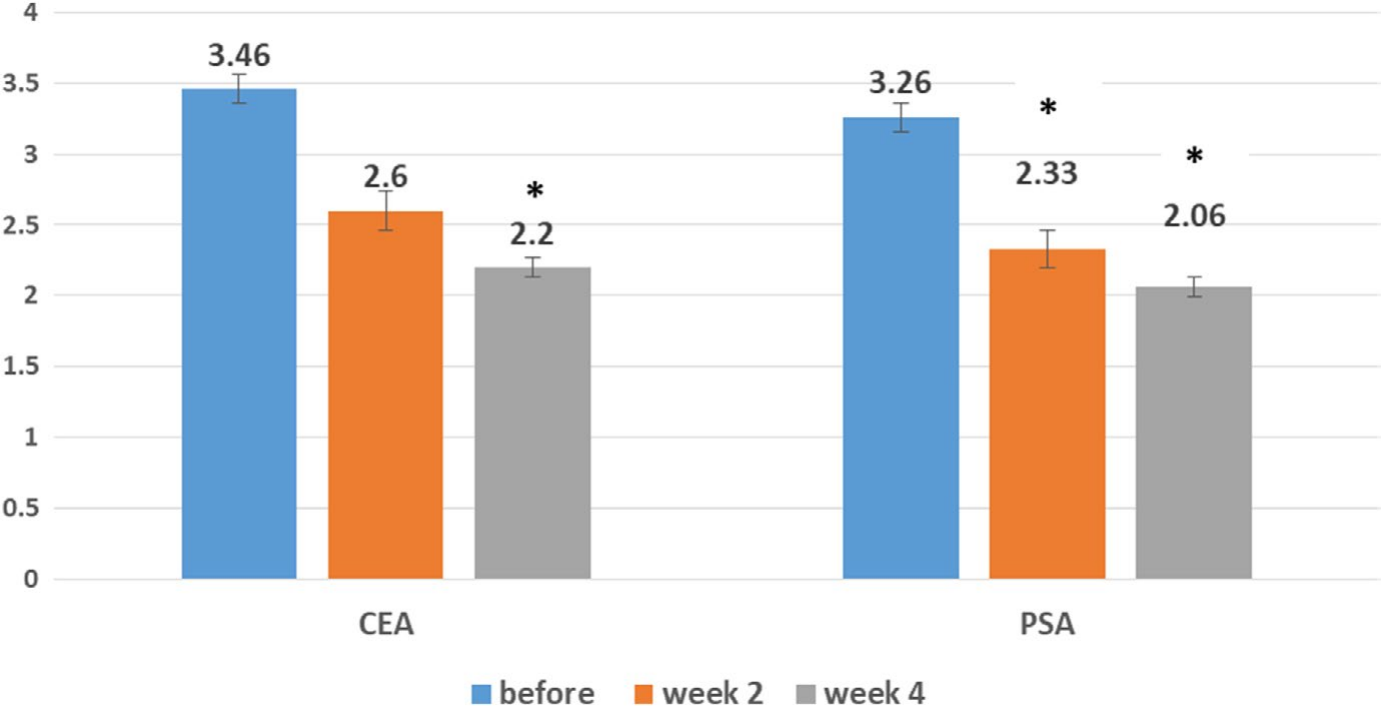
CEA decrease after 2 and 4 weeks, and PSA decrease after 2 and 4 weeks of treatment with oxymetazoline cream 1% in rosacea patients (*p‐value < 0.05).

The values of CEA and PSA indexes before, 2, and 4 weeks after the treatment are shown in Figure 4. At baseline, the mean CEA and PSA scores were 3.46 (SD: 0.51) and 3.26 (SD: 0.45), respectively. CEA decreased significantly after 2 weeks (mean: 2.60, SD: 0.50) and 4 weeks (mean: 2.20, SD: 0.41) of treatment, respectively (*p*‐value: 0.001, *p*‐value: 0.001). PSA decreased significantly after 2 weeks (mean: 2.33, SD: 0.48) and 4 weeks (mean: 2.06, SD: 0.25) of treatment, respectively (*p*‐value: 0.001, *p*‐value: 0.001).

No side effects of the medicine, such as dryness, itching, stinging, redness, scaling, or pigmentation changes, were reported.

## 4 Discussion

Recently, oxymetazoline hydrochloride entered the market with FDA approval for persistent erythema of rosacea. It appears to be safe and effective for its approved indication, with less rebound effect (1%) compared to the alternative available treatments. Adverse reactions have been observed in a small percentage of patients, most frequently in the first 3 months of treatment. Few patients discontinue its use, without worsening of the erythema [15]. However, its real‐world clinical potential and place among treatment guidelines and the potential of it to have a long‐term positive impact on patients, both symptomatically and psychologically, has remained unclear [16].

This study showed that oxymetazoline cream 1% can be effective in the treatment of rosacea patients, who generally have a darker skin type than their counterparts in Europe and North America, with improvements in skin biophysical parameters.

Erythema index decreased significantly at 2 and 4 weeks after the treatment. Erythema decrease was also seen in the patients' photos. Δ*E* and Δ*L*, which are calculated by CSI software and show the erythema and lightness difference between a point on the lesion and a point on the healthy skin, were improved as well. Comparing the results obtained from MPA 580 and Visoface devices and the significant reduction of erythema with both mexameter probe and Δ*E* index showed that the vasoconstriction and decrease of skin erythema with α_1_A adrenoceptor agonists in rosacea patients are confirmed by devices with different mechanisms. This efficacy has been also demonstrated by PSA and CEA assessments in different papers. Although CEA and PSA are not objective assessments, a significant reduction of them is of great importance, because it means improvement of rosacea symptoms from the physicians' and patients' perspectives, and their satisfaction with the treatment [17].

Efficacy assessment of oxymetazoline cream 1% for the treatment of persistent facial erythema associated with rosacea by Linda Stein‐Gold in 2018 showed at least a 2‐grade improvement in skin erythema from baseline on both CEA and PSA in 4 weeks [18]. The duration of oxymetazoline application and the significant reductions of CEA and PSA were the same in our study. The difference was the number of methods for evaluating efficacy, which was much more complete in this study.

Two other studies which examined the use of oxymetazoline cream 1% in adult patients with moderate to severe rosacea, by the method of CEA and PSA evaluation, showed 1 to 2° improvement in erythema [19, 20]. The difference was that our patients suffered from mild to moderate severity of disease and their evaluation methods didn't include objective techniques.

It was shown that long‐term (a full year) consistent daily use of oxymetazoline cream 1% resulted in one or more degrees of improvement in CEA and PSA criteria, and a robust and sustained response. This finding might provide clinicians with the evidence they need to encourage patients to continue the once‐daily application of oxymetazoline long‐term.

Capillaroscopic evaluations of rosacea lesions have demonstrated larger polygons, more prominent telangiectases, a larger mean vessel diameter, neoangiogenesis, and a reddish background, due to subpapillary vessel dilation [17]. Improvement of almost all of these changes was seen after treatment with oxymetazoline cream 1%.

Oxymetazoline cream significantly reduced stratum corneum hydration after 2 weeks, which could be due to the contraction of blood vessels and reduced blood flow [21], but it almost returned to its original value after 4 weeks of treatment. It showed that oxymetazoline cream 1% eventually didnot cause dry skin.

The pH, TEWL, and skin temperature values did not change significantly and remained almost constant, which indicated that the product did not harm the skin barrier status [22].

Enlargement of the sebaceous glands is usually seen in rosacea and is sometimes accompanied by an increase in sebum content [23]. The amount of sebum decreased (not significant) in the course of this study, which shows that this cream could be effective in this context.

An increase in epidermal and dermal density and a decrease in epidermal and dermal thickness were observed to some extent. This could be due to the reduction of inflammation and edema as a result of reduced interstitial fluid and outflow of blood from the arteries [24]. Oxymetazoline also inhibits neutrophil phagocytosis and oxidative reactions, thereby it reduces the production of inflammatory cytokines and inflammation [16].

From the absence of any significant reduction in melanin content, it can be concluded that oxymetazoline cream 1% does not have a hyperpigmentation side effect. Moreover, no specific side effects of the drug were observed in the patients. This finding is consistent with the clinical studies of the brand manufacturer, which established the long‐term safety and skin tolerance of daily use of oxymetazoline cream 1%, and no signs of recurrence have been observed [11].

This is one of the most comprehensive skin biometric evaluations—as an exact and objective method—to study the effect of oxymetalozine cream 1% on erythema of rosacea. The limitation of the study could be the low sample size, but all the expected improvements were seen during the trial with this sample size.

## 5 Conclusion

The results of this study showed that oxymetazoline cream 1% was safe and effective in the treatment of mild to moderate facial erythema of rosacea, according to various skin biometric assessments, as exact and objective evaluating methods. So it is recommended for improving erythema of rosacea, clinically confirmed with skin biometric assessments.

## Author Contributions

S.A.N., A.F., M.K. conceived the study, designed the protocol, and supervised data collection. A.S., F.S., M.A., F.A. conducted the statistical analysis and interpreted the results. F.S. managed patient recruitment and data acquisition. T.Y., A.N. drafted the manuscript and coordinated revisions. S.A.N., F.S. provided critical revisions for intellectual content. S.A.N. oversaw ethical compliance and regulatory submissions.

## Conflicts of Interest

Alireza Firooz and Saman Ahmad Nasrollahi are shareholders of Janus Pharmaceutical Co, Tehran, Iran. The other authors declare no conflicts of interest.

## Data Availability

Data available on request from the authors.

## References

[1] T. Dirschka , G. Micali , L. Papadopoulos , J. Tan , A. Layton , and S. Moore , “Perceptions on the Psychological Impact of Facial Erythema Associated With Rosacea: Results of International Survey,” Dermatology and Therapy 5, no. 2 (2015): 117–127. 26022994 10.1007/s13555-015-0077-2 PMC4470961

[2] M. Schaller , L. Almeida , A. Bewley , et al., “Recommendations for Rosacea Diagnosis, Classification and Management: Update From the Global ROSacea COnsensus 2019 Panel,” British Journal of Dermatology 182, no. 5 (2020): 1269–1276. 31392722 10.1111/bjd.18420 PMC7317217

[3] J. Tan , L. Almeida , A. Bewley , et al., “Updating the Diagnosis, Classification and Assessment of Rosacea: Recommendations From the Global ROSAcea CONsensus (ROSCO) Panel,” British Journal of Dermatology 176, no. 2 (2017): 431–438. 27718519 10.1111/bjd.15122

[4] J. Buddenkotte and M. Steinhoff , “Recent Advances in Understanding and Managing Rosacea,” F1000Research 7 (2018), 10.12688/f1000research.16537.1. PMC6281021 30631431

[5] A. D. Holmes and M. Steinhoff , “Integrative Concepts of Rosacea Pathophysiology, Clinical Presentation and New Therapeutics,” Experimental Dermatology 26, no. 8 (2017): 659–667. 27376863 10.1111/exd.13143

[6] J. Q. Del Rosso and E. Tanghetti , “Topical Oxymetazoline Hydrochloride Cream 1% for the Treatment of Persistent Facial Erythema of Rosacea in Adults: A Comprehensive Review of Current Evidence,” Journal of Clinical and Aesthetic Dermatology 14, no. 3 (2021): 32–37. PMC8021401 33841614

[7] M. Heisig and A. Reich , “Psychosocial Aspects of Rosacea With a Focus on Anxiety and Depression,” Clinical, Cosmetic and Investigational Dermatology 11 (2018): 103–107. 29551906 10.2147/CCID.S126850 PMC5844253

[8] F. Dall'Oglio , M. R. Nasca , and G. Micali , “Emerging Topical Drugs for the Treatment of Rosacea,” Expert Opinion on Emerging Drugs 26 (2021): 1–11. 33596752 10.1080/14728214.2021.1887138

[9] N. K. Nagrani , A. M. Gonzalez , P. M. Zito , B. P. Glick , and R. M. Rubenstein , “Topical Oxymetazoline (Rhofade) in Rosacea,” Journal of the Dermatology Nurses' Association 12, no. 5 (2020): 243–245.

[10] A. W. Kuang , J. DuBois , M. Attar , and G. Ahluwalia , “Clinical Pharmacokinetics of Oxymetazoline Cream Following Topical Facial Administration for the Treatment of Erythema Associated With Rosacea,” Journal of Drugs in Dermatology 17, no. 2 (2018): 213–220. 29462230

[11] Z. D. Draelos , M. H. Gold , R. A. Weiss , et al., “Efficacy and Safety of Oxymetazoline Cream 1.0% for Treatment of Persistent Facial Erythema Associated With Rosacea: Findings From the 52‐Week Open Label REVEAL Trial,” Journal of the American Academy of Dermatology 78, no. 6 (2018): 1156–1163. 29409914 10.1016/j.jaad.2018.01.027

[12] T. Yazdanparast , K. Yazdani , P. Humbert , et al., “Comparison of Biophysical, Biomechanical and Ultrasonographic Properties of Skin in Chronic Dermatitis, Psoriasis and Lichen Planus,” Medical Journal of the Islamic Republic of Iran 32 (2018): 108–634. 30815403 10.14196/mjiri.32.108 PMC6387801

[13] S. Cannavò , F. Guarneri , R. Giuffrida , E. Aragona , and C. Guarneri , “Evaluation of Cutaneous Surface Parameters in Psoriatic Patients,” Skin Research and Technology 23, no. 1 (2017): 41–47. 27270565 10.1111/srt.12299

[14] T. Yazdanparast , K. Yazdani , P. Humbert , et al., “Biophysical Measurements and Ultrasonographic Findings in Chronic Dermatitis in Comparison With Uninvolved Skin,” Indian Journal of Dermatology 64, no. 2 (2019): 90–96. 30983602 10.4103/ijd.IJD_464_17 PMC6440184

[15] C. M. M. de Oliveira , L. M. C. Almeida , R. R. Bonamigo , C. W. G. de Lima , and E. Bagatin , “Consensus on the Therapeutic Management of Rosacea‐Brazilian Society of Dermatology,” Anais Brasileiros de Dermatologia 95, no. s1 (2020): 53–69, 10.1016/j.abd.2020.08.001. 33172727 PMC7772594

[16] N. U. Patel , S. Shukla , J. Zaki , and S. R. Feldman , “Oxymetazoline Hydrochloride Cream for Facial Erythema Associated With Rosacea,” Expert Review of Clinical Pharmacology 10, no. 10 (2017): 1049–1054. 28837365 10.1080/17512433.2017.1370370

[17] J. Logger , F. de Vries , P. J. van Erp , E. de Jong , M. Peppelman , and R. Driessen , “Noninvasive Objective Skin Measurement Methods for Rosacea Assessment: A Systematic Review,” British Journal of Dermatology 182, no. 1 (2020): 55–66. 31120136 10.1111/bjd.18151

[18] L. Stein‐Gold , L. H. Kircik , Z. D. Draelos , et al., “WITHDRAWN: Efficacy and Safety of Topical Oxymetazoline Cream 1.0% for Treatment of Persistent Facial Erythema Associated With Rosacea: Findings From the 2 Phase 3, 29‐Day, Randomized, Controlled REVEAL Trials,” Journal of the American Academy of Dermatology Elsevier, (2018), 10.1016/j.abd.2020.08.001. 29409915

[19] E. A. Tanghetti , J. S. Dover , D. J. Goldberg , et al., “Clinically Relevant Reduction in Persistent Facial Erythema of Rosacea on the First Day of Treatment With Oxymetazoline Cream 1.0,” Journal of Drugs in Dermatology 17, no. 6 (2018): 621–626. 29879249

[20] J. DuBois , J. S. Dover , T. M. Jones , R. A. Weiss , D. R. Berk , and G. Ahluwalia , “Phase 2 Randomized, Dose‐Ranging Study of Oxymetazoline Cream for Treatment of Persistent Facial Erythema Associated With Rosacea,” Journal of Drugs in Dermatology 17, no. 3 (2018): 308–316. 29537449

[21] D. Antonov , S. Schliemann , and P. Elsner , “Methods for the Assessment of Barrier Function,” Skin Barrier Function 49 (2016): 61–70. 10.1159/000441546 26844898

[22] T. Yazdanparast , K. Yazdani , P. Humbert , et al., “Biophysical and Ultrasonographic Changes in Lichen Planus Compared With Uninvolved Skin,” International Journal of Women's Dermatology 5, no. 2 (2019): 100–104. 10.1016/j.ijwd.2018.10.001 PMC6451735 30997382

[23] M. Picardo , L. F. Eichenfield , and J. Tan , “Acne and Rosacea,” Dermatology and Therapy 7, no. 1 (2017): 43–52. 28150107 10.1007/s13555-016-0168-8 PMC5289119

[24] T. Yazdanparast , K. Yazdani , S. A. Nasrollahi , et al., “Biophysical and Ultrasonographic Changes in Pityriasis Rosea Compared With Uninvolved Skin,” International Journal of Women's Dermatology 7 (2020): 331–334. 10.1016/j.ijwd.2020.10.002 PMC8243123 34222592

